# Mitochondrial Dysfunction in Aging and Cancer

**DOI:** 10.3390/jcm8111983

**Published:** 2019-11-15

**Authors:** Loredana Moro

**Affiliations:** Institute of Biomembranes, Bioenergetics and Molecular Biotechnologies, National Research Council, 70126 Bari, Italy; l.moro@ibiom.cnr.it; Tel.: +39-080-544-9807

**Keywords:** mitochondrial DNA, mitochondria-to-nucleus signaling, aging, cancer

## Abstract

Aging is a major risk factor for developing cancer, suggesting that these two events may represent two sides of the same coin. It is becoming clear that some mechanisms involved in the aging process are shared with tumorigenesis, through convergent or divergent pathways. Increasing evidence supports a role for mitochondrial dysfunction in promoting aging and in supporting tumorigenesis and cancer progression to a metastatic phenotype. Here, a summary of the current knowledge of three aspects of mitochondrial biology that link mitochondria to aging and cancer is presented. In particular, the focus is on mutations and changes in content of the mitochondrial genome, activation of mitochondria-to-nucleus signaling and the newly discovered mitochondria-telomere communication.

## 1. Introduction

Mitochondria are cellular organelles that play a pivotal role in maintaining cellular homeostasis by regulating energy metabolism, cell survival and proliferation. Production of adenosine triphosphate (ATP) and the generation of intermediate metabolites are the traditional functions ascribed to mitochondria. Within the mitochondria, the tricarboxylic acid cycle (TCA cycle) generates mitochondrial metabolites and reducing equivalents, in the form of reduced nicotinamide adenine dinucleotide (NADH) and flavin adenine dinucleotide (FADH2), which enter into the mitochondrial electron respiratory chain (ETC). ETC includes four membrane-bound protein complexes and catalyzes the oxidation of reducing equivalents using oxygen as the terminal electron acceptor. The electron transfer is coupled to the transfer of protons across the inner mitochondrial membrane to generate an electrochemical gradient (mitochondrial membrane potential) required both for ATP synthesis through the ATP synthase complex, and for efficient shuttling of proteins across the inner mitochondrial membrane. Electron leakage can arise along the ETC, leading to the formation of reactive oxygen species (ROS), important second messengers in aging, cancer and other physiopathological conditions. The intermediate metabolites generated in the TCA cycle are the precursors of lipids, carbohydrates, proteins and other macromolecules [1]. Mitochondria are able to migrate within the cell, fuse and divide through rapid fusion and fission processes and undergo turnover through mitophagy, a specialized form of autophagy. Mitochondrial dynamics is regulated by the large dynamin family of GTPases, enables mitochondrial recruitment to subcellular compartments that require more energy and is important for mitochondrial quality control and for communication with the cytosol and the nucleus (reviewed in [2]). Mitochondrial fusion begins with the joining of the mitochondrial outer membrane mediated by the mitofusin proteins Mfn1 and Mfn2, followed by fusion of the mitochondrial inner membrane catalyzed by OPA1. Fusion occurs similarly for the inner and outer membrane and involves a formation of interlocking coiled coils mediated by fusion proteins on the two membranes, followed by their fusion catalyzed by GTP hydrolysis [3].

The inner membrane fusion is dependent upon the mitochondrial membrane potential [4]. The mitochondrial fission is instead mediated by the GTPase DRP1, a protein that is recruited to the outer membrane where it oligomerizes and forms a spiral around the outer and inner membrane that causes fragmentation of the mitochondrion [5,6]. Disruption of the normal fusion/fission balance results in mitochondrial dysfunction [6].

Recent evidences point to the mitochondrial content and functionality in the regulation of nuclear gene expression by affecting transcription and translation, as well as alternative splicing mechanisms [7]. Hence, mitochondrial dysfunction leading to changes in nuclear gene expression may affect the risk of degenerative diseases, cancer and aging [7,8,9,10].

Aging is the most important risk factor for cancer. According to the National Cancer Institute (NCI), the median age of a cancer diagnosis is 66 years (https://www.cancer.gov/about-cancer/causes-prevention/risk/age). At first glance, aging and cancer may seem to be two opposite processes, the first implying a slow decline of cellular and organismal functions, the second providing fitness to the cells. In reality, these two processes are strictly interconnected and share common origins. Aging is associated with the progressive accumulation of genetic and cellular damage [11,12]. However, DNA damage may confer survival and growth advantages to certain cells, which can eventually thrive and evolve into a tumorigenic phenotype. Genetic damage affecting the functionality of mitochondria has been associated with both aging and cancer.

Mitochondria have been the focus of the aging research for decades, when Harman proposed the free radical theory of aging [13,14], then revisited by Alexeyev as the mitochondrial theory of aging [15]. Both theories hypothesize that ROS are the main determinants of the loss of cellular performance observed with increasing age, with the mitochondrial theory implying mitochondria as the main producers of ROS, and consequently, mitochondria as key drivers of aging. In the last decade, knowledge of these organelles has expanded, providing more connections between mitochondrial biology and the aging process [16]. Similarly to aging, changes in mitochondrial functionality have been strictly linked to cancer [17]. Indeed, several studies have shown that mutations in mitochondrial proteins encoded by either the mitochondrial or the nuclear genome may favor cancer development and progression [8,9,18,19]. This review provides an overview of the convergent and divergent roles that mitochondrial dysfunction may play in cellular processes linked to aging and cancer, with a specific focus on mitochondrial DNA, mitochondria-to-nucleus signaling and telomere shortening.

## 2. The Mitochondrial Genome

Mitochondria are equipped with their own genome, the mitochondrial genome (mtDNA), a circular, double-stranded DNA molecule of ~16.5 kb in humans, present in one to several thousand copies per cell, depending on the tissue and cell type. The two mtDNA strands have different resolution on a cesium chloride gradient and can be separated in a heavy strand (H-strand) rich in G and a light strand (L-strand) rich in C [20]. In addition, the mtDNA has a unique regulatory region, the displacement loop (D-loop), a triple-stranded region that includes a short single-strand DNA molecule, the 7S DNA [21]. The mitochondrial genome encodes for 13 proteins belonging to the respiratory complexes I, III, IV and V, and for two rRNAs and 22 tRNAs required for mitochondrial protein synthesis. Most of the mtDNA information is encoded by the H-strand (12 proteins, 2 rRNAs, 14 tRNAs). The mtDNA is maternally inherited and depends upon nuclear-encoded proteins for its replication and transcription. Replication of mtDNA is performed by DNA polymerase γ (POLγ), twinkle helicase and mitochondrial single-stranded binding protein (mtSSB), and proceeds throughout the entire life of an organism in both dividing and terminally differentiating cells. The exact molecular mechanism involved in the regulation of mtDNA replication in response to extracellular stimuli/stress remains a poorly investigated area [22]. The mitochondrial proteome is dynamic; i.e., the protein composition and the levels of a given protein may vary depending on the tissue and cell type. Even within the same cell type the mitochondrial proteome may vary over time depending on specific external stimuli or stress conditions [23]. 

Though the majority of the mammalian mitochondrial proteome (about 1200 proteins) is encoded by the nuclear genome and then imported into mitochondria, the 13 proteins encoded by the mtDNA (1% of the mitochondrial proteome) play an essential role for the mitochondrial function. Mutations or depletion of the mtDNA can indeed impair the energy metabolism and alter the intracellular signaling, resulting in mitochondrial dysfunction that may affect the cell performance at various degrees, leading to “mitochondrial diseases” in extreme cases [8,9,24,25]. MtDNA accumulates mutations at a considerably higher rate than nuclear DNA [26]. Traditionally, the increased mutational load was ascribed to a lack of efficient repair systems, lack of protective histones and to the close proximity of mtDNA to the respiratory chain complexes, the main site of production of ROS. Though mtDNA lacks traditional histones, the mitochondrial transcriptional factor A (TFAM) is tightly associated to the mtDNA molecules and plays an histone-like protective role [27]. However, mitochondria lack nucleotide excision repair (NER), efficient homologous recombination, microhomology-mediated end joining (MMEJ) and non-homologous end joining (NHEJ). The short and long patch base excision repair (BER) and single strand break repair pathways are instead preserved [28]. Hundreds of mtDNA mutations have been reported leading to a variety of human disorders (www.mitomap.org). In most cases, wild-type and mutated mtDNA coexist within the same cell, a condition known as heteroplasmy, as opposed to the term homoplasmy, when all the copies of the mtDNA are identical. The severity of the mtDNA-associated disorders is dependent on the level of heteroplasmy. For example, the mitochondrial dysfunction observed in the MELAS (mitochondrial myopathy, encephalopathy, lactic acidosis and stroke-like episodes) syndrome, usually caused by a mutation within the mtDNA-encoded tRNA Leu (UUR), can be rescued by levels of wild-type mtDNA above 6% [29]. In general, a pathogenic mtDNA mutation would need to reach a threshold level of more than 60% in a given tissue or cell type to produce a measurable bioenergetic defect [30].

### 2.1. MtDNA and Aging

MtDNA point mutations and deletions are known to accumulate with age in human tissues, including brain, heart and skeletal muscles [31,32,33,34]. A debate is still ongoing to define whether accumulation of mtDNA mutations is causal or just a correlation with aging. So far, the strongest evidence favoring the hypothesis of a causal role of mtDNA changes in aging comes from the generation of homozygous knock-in mice for the catalytic subunit of POLγ that lacks proofreading activity. These mice exhibit a mtDNA mutator phenotype, with accumulation of extremely high levels of mtDNA mutations, a shortened lifespan and early onset of age-associated phenotypes, including hair graying, weight loss, reduced subcutaneous fat, osteoporosis, alopecia and kyphosis [35,36]. Of note, while an increased load of mtDNA point mutations has not been linked to the aging phenotype [37], the presence of large mtDNA deletions has been reported as a driver of premature aging in mitochondrial mutator mice [38]. These mice apparently did not exhibit a net increase in ROS levels [35], and in general, in oxidative stress markers [36]. However, Kolesar et al. [39] have recently shown that the mitochondrial protein content in the muscle tissues of Polγ-knock-out mice is significantly reduced compared with wild-type mice, which results in a net increase in ROS levels per mitochondrial proteins, and a burst in mtDNA and mitochondrial protein oxidative stress. Increased oxidative stress in the muscle of Polγ-knock-out mice has been confirmed by a subsequent study [40]. These findings support previous experimental evidences showing an anti-aging role of mitochondria-targeted catalase, an anti-oxidant enzyme [41,42,43]. Further experiments in mice have shown that maternally inherited mtDNA mutations may cause premature aging signs, such as alopecia, kyphosis and premature death [44,45]. In humans, HIV patients treated with nucleoside analog reverse transcriptase inhibitor antiretroviral drugs have shown signs of premature aging, including increased cardiovascular disease and bone fractures [46]. Subsequent studies have shown that these antiretroviral drugs have mitochondria as off-target sites, and in particular, they inhibit POLγ, causing mtDNA mutations and depletion that result in mitochondrial dysfunction [47]. Another example linking mtDNA to the aging phenotype comes from human subjects carrying mutations in POLγ. More than 300 pathogenic mutations in human POLγ have been identified so far (https://tools.niehs.nih.gov/polg/), and they have been linked to several disorders, including age-related pathologies such as Parkinson’s disease [10,48,49].

Loss of mitochondrial fusion in the skeletal muscle of *Mfn1*- and *Mfn2*-knockout mice has been linked to increased mtDNA mutations and deletions, as well as mtDNA depletion. These molecular events precede the phenotypic changes observed in these mice, such as impaired mitochondrial functionality, abnormal mitochondria proliferation and muscle loss [50]. Overall, increased mitochondrial fusion may support longevity, as highlighted by a recent study in *C. elegans* [51]. In addition, a decline in mtDNA content has been observed in peripheral mononuclear blood cells (PMBC) during aging, and it has been linked to reduced immune response in the elderly [52,53]. Recently, a novel and very precise technique has allowed researchers to ascertain that healthy centenarians retain more mtDNA copies and less mtDNA deletions during immune cell stimulation than old people and frail centenarians [54], implying that the retention of a high amount of mtDNA is a hallmark of healthy aging.

### 2.2. MtDNA and Cancer

MtDNA mutations and changes in mtDNA content have been associated with cancer progression in a variety of cancer types (reviewed in [8,9]). A large-scale analysis performed on several cancer types of the TCGA dataset has recently confirmed that many, but not all cancers, display a significant depletion of the mtDNA content, which is directly associated with reduction in the expression of genes of the mitochondrial respiratory chain, and inversely correlated with the expression of genes of the immune response and cell cycle [55]. Some somatic mtDNA mutations were reported to favor tumorigenesis [56] and others to promote cancer metastasis [57] through an increase in ROS production [56,57]. However, a definitive link between mtDNA depletion/somatic mutations and tumorigenesis or cancer progression is still missing. Experiments in cell culture have shown that induced mtDNA depletion may restrain tumorigenesis [58,59] but support cancer cell invasion and metastasis [9,60,61,62,63,64]. Partial mtDNA depletion results in decreased mitochondrial membrane potential, increased DRP1 mitochondrial localization and decreased OPA1 levels, accompanied by increased mitochondrial fission, as demonstrated by enrichment in fragmented mitochondria [65]. In turn, increased mitochondrial fission alters the cytoskeleton and promotes the formation of pseudopodia-like structures, typical of invasive cells [65]. Consistently, increased mitochondrial fission has been frequently detected in carcinoma samples (reviewed in [66]). Analysis of the TCGA dataset reported a direct correlation between mtDNA depletion and reduced patients’ survival [55]. An independent study performed on 8161 normal and cancer samples in the TCGA dataset showed that decreased oxidative phosphorylation is associated with poor survival across multiple cancer types and with an epithelia-to-mesenchymal (EMT) gene expression profile [67], typical of cells with acquired invasive abilities [9]. Furthermore, analysis of the Skin Cutaneous Melanoma dataset containing 367 metastatic lesions and 103 primary cancer samples demonstrated that: (i) Reduced oxidative phosphorylation was the most significant metabolic signature in the metastatic lesions compared with the primary cancers; and (ii) EMT was strongly upregulated in the metastatic cancer samples [67], supporting a role of mitochondrial dysfunction in the metastatic process. A previous study performed on 49 patients with advanced metastatic melanoma has shown that stage IV melanomas may be dependent either on glycolysis or a combination of glycolysis and oxidative phosphorylation, which may suggest a metabolic symbiosis within the same tumor [68]. In agreement with this study, Najjar et al. [69] reported that metabolic adaptation can vary in melanoma samples, with some cells exhibiting only deregulated glycolysis and others only deregulated oxidative phosphorylation. Patient-derived melanoma cell lines with a prevalent oxidative metabolism showed resistance to immunotherapy as well [69]. Recent evidences have pointed out to the horizontal transfer of whole mitochondria from host cells present in the tumor microenvironment to respiratory-deficient tumor cells, depleted of mtDNA [70]. This transfer would recover mitochondrial respiratory activity and promote tumor growth [70]. Based on these observations, it is tempting to speculate that mtDNA depletion/deletions and pathogenic mtDNA mutations may confer a selective advantage to certain cancer cells for invading and thriving in hostile microenvironments, such as the circulating system, while abating cell proliferation. Partial reconstitution of the wild-type mtDNA pool by horizontal transfer from stromal cells would allow cancer cells to recover their proliferative capacity, and thus, their full tumorigenic potential.

## 3. Retrograde Signaling in Aging and Cancer

Besides being considered as the cell’s “energy hubs”, mitochondria have emerged as critical “signaling hubs” in regulating cell homeostasis [71]. Communication from mitochondria to the nucleus is known as “retrograde signaling”, opposed to the traditional anterograde signaling, i.e., the communication pathways from the nucleus to the mitochondria. It has been first described by Butow in yeast. That is, in yeast cells depleted of mtDNA, Butow described changes in the transcription of nuclear-encoded genes, and an increase in the transcription of genes associated with a metabolic shift from aerobic to anaerobic respiration (reviewed in [72]). Screenings performed in *C. elegans* have revealed that impairing the mitochondrial respiratory chain unexpectedly extends the lifespan [73]. This observation seems a paradox given that aging tissues are characterized by loss of mitochondrial fitness. Similarly, in *C. elegans* a transient increase of mitochondrial ROS triggers changes in gene expression that promote longevity instead of aging, sharply in contrast with the mitochondrial theory of aging [74]. These findings have been corroborated in mammals using mouse models. For example, mice heterozygous for Mclk1, a mitochondrial protein necessary for the biosynthesis of ubiquinone, show increased lifespan correlated with early impairment of the mitochondrial functionality, significant reduction of mitochondrial electron transport, ATP and NAD+ levels [75]. The reason why mitochondrial stress may counteract the aging process is still not completely understood. Some experimental evidences suggest that the retrograde signaling activated by the mitochondrial stress may play a critical role. In the context of aging, activation of the mitochondrial unfolded protein response (UPRmt) seems to be important in signaling a mitochondrial stress to the nucleus. This retrograde response is elicited by mtDNA depletion or by protein misfolding occurring in these mitochondria [76]. It was described for the first time in mammalian cells depleted of mtDNA through ethidium bromide treatment, which exhibited induction of mRNAs for mitochondrial proteases and chaperons [77]. A similar transcriptional profile was observed upon overexpression in the mitochondrial matrix of a dominant negative ornithine transcarbamylase, an enzyme essential for protein processing and folding, suggesting a link between mitochondrial dysfunction, alteration of proteostasis mechanisms and the activation of UPRmt [78]. Work in *C. elegans* and other organisms has identified several components of this retrograde response (for a comprehensive review, see [79]). In *C. elegans*, mitochondrial dysfunction reduces the mitochondrial import efficiency of the transcription factor ATFS-1, allowing it to accumulate in the nucleus where it induces the transcription of hundreds of genes, including antioxidant proteins, proteases and enzymes involved in cellular metabolism that would promote survival and recovery of the mitochondrial functionality, thus supporting longevity and lifespan [78]. However, prolonged UPRmt activation occurring in the context of a heteroplasmic mtDNA pool seems to exacerbate mitochondrial dysfunction because it would result in an accumulation of damaged mtDNA (for a detailed review see [76]).

Evidence is accumulating on the important role that mitochondrial metabolites may play as second messengers eliciting epigenetic changes in the nucleus (for comprehensive reviews see [80,81]). In this context, acetyl-CoA is one of the most studied signaling molecules. This metabolite is present in mitochondria, cytosol and the nucleus. In mitochondria, it can be produced through different metabolic pathways, including conversion of pyruvate to acetyl-CoA by the pyruvate dehydrogenase complex (PDH), fatty acid β-oxidation, amino acid metabolism, direct synthesis by a reaction of ligation of acetate with CoA catalyzed by mitochondrial acyl-CoA synthetase short-chain family member 1 (ACSS1). Mitochondrial acetyl-CoA enters into the TCA cycle together with oxaloacetate to produce citrate, which is oxidized, allowing production of ATP through the oxidative phosphorylation. 

Once inside mitochondria, acetyl-CoA is unable to cross the inner mitochondrial membrane. To replenish its cytosolic and nuclear pool, citrate exported from mitochondria via the tricarboxylate carrier is converted to acetyl-CoA and oxaloacetate by the ATP-citrate lyase (ACLY) [82]. Cytosolic acetyl-CoA can participate to biosynthetic pathways, such as to fatty acids synthesis, and provides acetyls for lysine acetylation of proteins, thereby regulating their activity and localization [83]. In the nucleus, protein lysine acetylation modulates histone acetylation. It has been recently reported that the PDH complex can translocate from mitochondria to the nucleus during active cell proliferation, generating a nuclear pool of acetyl-CoA for fine-tuning histone acetylation [84]. Acetyl-CoA may thus function as a “sentinel metabolite”, where under stress conditions, acetyl-CoA would be diverted to mitochondria to provide energy and mitochondria-generated products, such as ketone bodies. Instead, elevated nucleo-cytosolic levels of acetyl-CoA would signal permissive conditions for growth, biosynthesis of lipids and histone acetylation. Indeed, a recent work has reported that high cytosolic/nuclear levels of acetyl-CoA due to overexpression of ACLY promote pancreatic cancer development [85]. In aging cells, elevated nucleo-cytosolic acetyl-CoA levels function as the metabolic repressor of autophagy [86]. Consistently, brain-specific knock-down of acetyl-CoA synthetase increases lifespan in *Drosophila* [86], suggesting that mitochondrial metabolites may play opposite roles in aging and cancer.

Chronic inflammation has been linked to aging and age-related pathologies, as well as to cancer (for detailed reviews see [87,88]). The inflammation process represents an essential immunological defense system that promotes survival. It has been extensively reported that mitochondrial dysfunction can promote the release of factors, known as damage-associated molecular patterns (DAMPs), into the cytosol, which can trigger an inflammatory response by engaging pattern recognition receptors. Mitochondrial DAMPs (mtDAMPs) include several molecules/effectors, such as mtDNA and mitochondria-produced ROS, which can activate the inflammasomes, multi-protein cytosolic complexes consisting of the adaptor apoptosis-associated speck-like protein (ASC), the inflammatory cysteine protease caspase-1, NOD-, LRR- and pyrin domain-containing protein 1 (NLRP1), NLRP3, NOD-, LRR- and CARD-containing protein 4 (NLRC4), and absent in melanoma 2 (AIM2). Among different inflammasomes, mitochondrial stress has been implicated in the regulation of NLRP3 activity, with mtDNA released into the cytosol as a triggering DAMP of NLRP3 inflammasome. In macrophages, cytosolic accumulation of mtDNA upon stress activates caspase-1 and promotes the secretion of IL-1β and IL-18 through activation of the NLRP3 inflammasome [89]. This retrograde response requires mitochondria-generated ROS that would cause a release of mtDNA into the cytosol [89]. Degraded mtDNA has been shown to induce the release of pro-inflammatory cytokines in brain cells and represents a possible trigger of neurodegenerative processes [90]. Recent reports further support the role of mitochondrial ROS and mtDNA in the activation of NLRP3 in age-dependent diseases, including atherosclerosis [91,92,93,94,95,96], as well as in cancer, though the activation of inflammasomes during tumor development and progression still remains controversial and with conflicting outcomes (for a detailed review see [97]). Recent evidences point to a role of DAMPs released by dying cancer cells upon chemotherapy and inflammasome’s activation in bursting an immune response that could help the removal of the remaining live cancer cells. In this context, it has been proposed that a particular class of mitocans (mitochondrial-targeted anti-cancer drugs), like vitamin E analogs, that selectively induce cell death by triggering ROS production in cancer cell mitochondria, may be promising candidates for anti-cancer therapy (reviewed in [98]).

Work from Avadhani’s group has shown that mammalian cells can activate another mitochondria-to-nucleus signaling pathway upon mitochondrial respiratory stress [72,99]. This signaling pathway considers Ca^2+^ as the main second messenger involved in the transmission of stress signals from mitochondria to nucleus and has been related to the response to a mitochondrial stress in the context of cancer cells. Mitochondria store large amounts of Ca^2+^. Upon mitochondrial stress/dysfunction leading to reduced mitochondrial membrane potential, Ca^2+^ is released into the cytosol where it can activate Ca^2+^-dependent signaling. An early event of this signaling is activation of the phosphatase Calcineurin. This Ca^2+^/calcineurin-mediated pathway can be ROS-dependent or -independent, in relation to the cell type. For example, it is ROS-independent in skeletal myocytes, but ROS-dependent in macrophages [60,100,101]. In turn, the Ca^2+^/calcineurin signaling would activate the insulin-like growth factor receptor I (IGF1R) in a growth factor-independent manner that, by promoting the PI3-kinase/AKT pathway, would culminate in the induction of the transcription of nuclear genes (reviewed in [65,99]). Ca^2+^ may also activate other pathways independently on calcineurin. From a “phenotypic” point of view, Ca^2+^-mediated signaling upon mitochondrial stress results in survival, resistance to pro-apoptotic drugs, increased glycolysis and invasion of otherwise not invasive cells [8,9,61,63,99,102,103]. Taken together, these experimental evidences are consistent with a role of mild mitochondrial dysfunction in supporting health and fitness of the cells, both in aging and in cancer, through the activation of a protective retrograde signaling.

## 4. Telomeres in Aging and Cancer

Telomeres are DNA-protein structures present at both ends of each chromosome that protect the genome from interchromosomal fusion and rapid nucleolytic degradation. These specialized structures function as biological clocks: most normal cells have a limited lifespan and at each cell division, telomeres lose a portion of DNA. After a certain number of cell divisions, telomeres reach a critical minimal length and cells undergo apoptosis [104]. Telomere length decreases with the aging process and the rate of telomere shortening may indicate the pace of aging [104]. Stem cells and cancer cells express high levels of telomerase, an enzyme known as hTERT in humans (human TElomerase Reverse Transcriptase), which is able to prevent telomeres’ shortening by elongating the telomeres’ DNA. Most somatic cells instead do not express telomerase. In addition to its established role in regulating telomere length in the nucleus, TERT exerts a protective role within mitochondria by shuttling from the nucleus to the mitochondria upon exogenous stress [105,106,107,108,109]. Within mitochondria, TERT binds mtDNA and protects it from damage, thus preserving the mitochondrial function and promoting resistance to apoptosis in cancer cells [108,109,110]. Consistently, expression of a mutant telomerase lacking a nuclear export signal inhibits cancer cell proliferation, prevents immortalization, promotes mitochondrial dysfunction and sensitivity to ionizing radiation and hydrogen peroxide exposure, two genotoxic stresses [111,112,113].

Inherited mutations in genes encoding for protein components of the telomeres cause accelerated aging symptoms (cardiovascular disease, hair graying, diabetes, poor immune response) [114]. Interestingly, in a yeast model system, lack of telomerase accelerated aging independently on de-protected telomeres [115]. Overexpression of telomerase can delay aging but would increase the risk of tumor formation [116]. Consistently, 80%–90% of malignant tumors show overexpression of hTERT, which may occur via multiple mechanisms, including epigenetic modifications (h*TERT* gene promoter methylation), h*TERT* amplification or structural variants (reviewed in [117]). Overall, up-regulation of hTERT confers unlimited replicative potential to cancer cells, and is one of the distinctive hallmarks of cancer [118].

Despite overexpression of hTERT in cancer cells, increasing evidence demonstrates that telomere shortening correlates with cancer aggressiveness (reviewed in [119]), suggesting a threshold of telomere shortening in cancer. In this context, hTERT may guarantee an optimal telomere length that would prevent replicative senescence. In addition, expression of hTERT splice variants in cancer cells has been shown to confer a growth advantage and resistance to chemotherapeutic drugs independently upon telomere protection [120], supporting the notion that hTERT plays multiple roles in different pathophysiological conditions.

A recent work has shown a direct connection between mitochondrial dysfunction and telomere attrition [121]. Qian et al. have used a chemoptogenetic approach to produce short-lived singlet oxygen (a highly reactive ROS) in the mitochondrial matrix, whose destiny in the whole cells may be easily monitored. One pulse of singlet oxygen produced secondary ROS (superoxide radical anion and hydrogen peroxide) that promoted mtDNA damage, decrease in mitochondrial respiration and cell cycle arrest. Intriguingly, these secondary ROS were detected in the nucleus where they induced telomere fragility and loss, without causing general nuclear DNA strand breaks. DNA double-strand breaks were exclusively present in telomeres. In turn, telomere dysfunction promoted Ataxia-Telangiectasia Mutated (ATM)-dependent repair of DNA damage, preventing cells from undergoing apoptosis [121]. These findings highlight a novel mitochondria-telomere axis activated by mitochondrial ROS and associated with mitochondrial dysfunction that may be important both in aging and in cancer. Future studies are needed to assess whether this new retrograde signaling pathway may also activate hTERT, thus supporting cell survival in the long term, and tumorigenesis. Alternatively, since in progeroid syndrome this telomere dysfunction has been shown to impair the mitochondrial function [122], it is possible that an initial, transient mitochondrial dysfunction may cause progressive loss of cell fitness and apoptosis via induction of telomere shortening in a vicious cycle.

## 5. Conclusions

**Figure 1:**
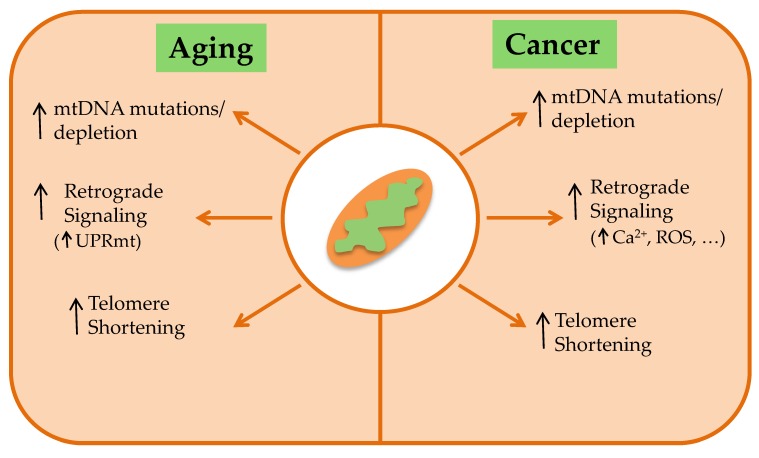
Mitochondrial dysfunction in aging and cancer. Aging and cancer share common mechanisms that include an alteration of the mitochondrial genome (mtDNA), activation of mitochondria-to-nucleus signaling pathways (retrograde signaling; see text for details) and telomere shortening. The latter is the biological clock of the cells, and beyond a limit, would cause cell cycle arrest and apoptosis, preventing the unlimited replication of normal cells. In cancer cells, expression of the telomerase hTERT would prevent excessive shortening of telomeres. Recent evidence points to mitochondrial dysfunction as a regulator of telomere shortening.

Mitochondrial function is critical for cell homeostasis, and alteration of mitochondrial performance is linked both to aging and cancer (Figure 1), two opposite processes, being aging associated with “loss of function” and cancer with “gain of fitness”.

MtDNA mutations and/or decreased mtDNA content have been described in aging tissues and cancer cells. In aging, they have been associated with reduced respiration and available energy, thus decreasing the rate of anabolic reactions for biosynthesis of the cell’s bricks (lipids, proteins, carbohydrates) and leading to physiological decline. Genetic studies in mouse models have provided evidence for a causal link between mtDNA depletion and the aging symptoms [35,44,45]. In cancer, some mtDNA point mutations seem to favor tumorigenesis, and other mutations would promote cancer metastasis, while mtDNA depletion correlates with poor patients’ survival in certain cancers. Though there are several evidences in support of the causal role of mtDNA changes in cancer, a definitive link between mtDNA depletion/somatic mutations and tumorigenesis/cancer progression is still missing.

Intriguingly, mouse models with a mtDNA mutator phenotype that display progeroid syndrome do not exhibit increased production of mitochondrial ROS [35], in contradiction with the core hypothesis of the mitochondrial theory of aging. In cancer, increase in ROS elicited by certain mtDNA mutations favors tumorigenesis and metastasis [56,57]. Recent studies have highlighted the role of alternative retrograde signaling transmitted by dysfunctional mitochondria to the nucleus that may modulate aging and cancer. 

These signals include the UPRmt, relevant to the aging process, and Ca^2+^-mediated signaling pathways, involved in the acquisition of some hallmarks of cancer cells. Mitochondrial dysfunction can also trigger telomere fragility and shortening, a common trait of aging and cancer. However, at variance with the aging process, cancer cells would not undergo cell cycle arrest because of the genetic/epigenetic upregulation of telomerase. These pathways are not mutually exclusive; rather they may intersect and contribute to the phenotype. Future studies, aimed at understanding the relative contribution of different mitochondrial retrograde signaling to aging and cancer, may open the road to the development of biologically active molecules targeting specific retrograde signaling pathways, in order to delay progression of age-related symptoms and/or to inhibit tumor growth and metastasis.
